# Visual pathways functioning in healthy pre-term adolescents: Sex but not gestational age effect

**DOI:** 10.1038/s41390-024-03513-9

**Published:** 2024-10-16

**Authors:** Matilde Taddei, Francesca Tinelli, Flavia Faccio, Daria Riva, Sara Bulgheroni

**Affiliations:** 1 Pediatric Neuroscience Department Fondazione IRCCS Istituto Neurologico Carlo Besta, Milano, Italy; 2 Department of Developmental Neuroscience IRCCS Fondazione Stella Maris, Pisa, Italy

## Abstract

**Background:**

Visuo-spatial and visuo-perceptual functioning is widely studied in preterm child and is strongly sex-specific. However, little to no data is available regarding male-female differences in preterm children and adolescents and about the interaction effect between sex and preterm birth.

**Methods:**

We studied 30 adolescents born preterm with normal cognitive and clinical neurological outcomes and 34 age-matched controls to investigate the interaction between levels of prematurity and sex in predicting the outcome of visual pathways functioning and to explore the relation between psychophysiological perceptive processing and neuropsychological performance.

**Results:**

In the presence of prematurity, a greater female vulnerability in central visuo-cognitive processing (Form Coherence Task), but not in neuropsychological accuracy (Street Completion Test and Visual Object and Space Perception battery), seems to be more evident. Moreover, the psychophysical threshold is correlated to neuropsychological accuracy only in preterm females and not in males.

**Conclusion:**

These results support the idea that the male vulnerability in cognitive functioning described in prematurity-related developmental conditions is negligible during school age in children-adolescents with normal cognitive and clinical neurological outcomes.

**Impact:**

- Visuo-perceptual functioning is widely studied in prematurity. However, few data are available about the interaction effect between sex and preterm birth in predicting visuo-perceptual functioning.
- We evidenced that in females born preterm with preserved cognitive abilities, the efficiency of the psychophysical visuo-perceptual threshold is reduced, but not related to the neuropsychological performance. Females may implement compensation strategies to achieve good performance regardless of the perceptual threshold.
- The present study addresses an important gap in literature, suggesting possible sex-specific outcomes in visuo-perceptual ability among preterm children and adolescents with normal intelligence and neurological outcomes.

## Introduction

Literature on developmental outcomes of very premature children, i.e. born before the end of 32 weeks of gestational age, provides evidence of high vulnerability to impairment in cognitive and neuropsychological abilities.^1,2^ While there is consistent evidence that low birthweight and premature birth have lasting effects on cognition, it is not clear whether males or females may be more susceptible to such effects. The so-called “male disadvantage hypothesis” describes a higher risk of developing prematurity-related diseases in males and unfavorable cognitive-behavioral outcomes for boys in the early stages of development.^3,4^ This trend seems to become less evident over time^5,6^ and, during school age, both the environmental and neurobiological risks, but not sex-related risks, seem to be consistent in predicting preterm variability in the neuropsychological outcomes.^3^ Recently, a systematic review and meta-analysis of several studies about this issue, concluded for no significant evidence that males and females differ in their susceptibility to the effects of severe or moderate prematurity/low birthweight on cognitive function, internalizing traits or externalizing traits.^7^

Visuo-perceptual processing has been extensively studied in preterm-born population, considering the importance of early visual function for the early development of sensory-motor and cognitive skills, which can be tracked from early childhood until school age and adulthood.^8,9^ These investigations are often implemented in the framework of the two cortical visual pathways implied in processing visual information – the dorsal stream and the ventral stream– involved in spatial aspects of visual perception and in object recognition according to size, shape, orientation, and color.^10–12^ Atkinson and Braddick^10^ suggested that the cluster of deficits seen until school age in children born preterm (spatial, motor, attention, and executive function) may be related to networks involving the cortical “dorsal” stream and its connections to parietal, frontal and hippocampal areas.^10^

Other studies explored whether a visuo-spatial deficit could be partially related to prematurity per se and not only to the higher risk of retinal damage or brain injuries at the level of periventricular white matter in preterm infants.^9^ Pavlova and colleagues^13^ addressed whether early disorders in the production of biological movement corresponded to an impairment in motion perception in preterm children. Results showed that the sensitivity to biological motion was related to the extent of periventricular leukomalacia over the parieto-occipital complex, and not to the severity of the motor disorder, confirming that motor experience per se does not appear to be necessary for the visual analysis of human movement.

Guzzetta et al.^14^ provided the first investigation of both movement and form perception with different stimuli (pure global translation/rotation motion, motion with some form information, and form-defined static) in premature children; results indicated that the dorsal stream-related functions (motion processing) are impaired in premature children irrespective of the presence of brain damage, whereas deficits in the ventral stream (form perception) are more related to the presence of periventricular brain damage. More recently, Benassi and collaborators^15^ confirmed a prevalent deficit in motion rather than form perception in preterm children when correcting for Intelligence Quotient (IQ) and visual acuity. No association was found between motion perception accuracy and gestational age, previous retinopathy of prematurity, or previous intraventricular hemorrhage in the early preterm group. Mathewson and collaborators^8^ argued that the deficits in visual discrimination described in extremely premature birth survivors compared to full-term born adults may be underpinned by decrements in Performance IQ and cognitive processing speed secondary to extremely preterm birth.

These data confirm the importance of controlling for potentially confounding clinical variables and studying children and adolescents without sensory, neurological, and intellectual sequelae of prematurity when investigating the visuo-perceptual and visuo-spatial domains and their related brain networks. The need to control for preterm neurological damages is particularly relevant when addressing sex specificity in neuro-cognitive sequelae of prematurity, to separate the effect of neurological injuries consequent to preterm birth from the effect of the interaction between sex and preterm birth. Thus, it would be good practice to investigate male-female differences either stratifying preterm children and adolescents at high and low neurological risk or controlling for neurological risks. Moreover, to compare emerging sex differences in the premature population with those observed in full-term born children could help to discriminate sex-related differences unspecific to the preterm child, but simply reflecting what can already be observed in the general population. Of notice, these methodological premises are even more critical when investigating visuo-spatial and visuo-perceptual abilities that are often described to be sex-specific in the general population with an advantage for males.^16,17^

Despite visuo-cognitive domain is frequently investigated in preterm literature and several evidence talk about sex specificity of this domain in general population, studies investigating whether sex differences in visuo-spatial and visuo-perceptual domains are present also in preterm population are still lacking, as we recently evidenced in a review on the argument.^18^ The very few data available are inconsistent and describe alternatively a worsen outcome in boy,^19,20^ or in girls^1^ or no sex differences^21^ in visual-spatial and visuo-motor skills, despite the detrimental effect of extreme prematurity - more common in boys - is not always controlled.^19^

In order to give some insight on how sex differences influence the visuo-perceptual and visuo-spatial outcome in very preterm children, we evaluated data collected on preterm adolescents in the light of sex differences. This is a reprocessing of data from our original project oriented to investigate disorders of visual perception in preterm children at different ages.^11^ Results published in a previous manuscript showed that preterm subjects had the same performance on all “ventral” tasks with respect to full-term controls, without any correlation with gestational age or birth weight. A statistically significant difference was instead found between preterm children and controls in two tasks of the VOSP battery that mostly involve the dorsal stream. These findings showed that preterm birth per-se (in absence of evident brain lesions) is not sufficient to compromise the development of the ventral pathway.^11^

For the aim of the present study, we have analyzed sex differences in the same 30 preterm adolescents and 34 age-matched controls, to investigate the interaction between levels of prematurity and sex in predicting the outcome of visual pathways functioning in adolescence. In particular, we hypothesize that the male disadvantage hypothesis would be less marked when evaluating the visual-spatial domain in school-age preterm-born children without clinical neurological sequaele. Ad interim results have been published in the context of a review about sex influences in the outcome of prematurity.^18^ In the present manuscript, we extended the analyses to further investigate whether the relation between psychophysiological perceptive processing and neuropsychological performance in the viso-cognitive domain may be modulated by sex and different levels of prematurity.

## Methods

The specific procedures about participant recruiting, inclusion criteria, statistical sample size a-priori power analysis, informed consent, ethical approval and detailed description of the test administered to patients and controls are provided elsewhere,^11^ but we briefly report the most important information below.

### Participants

Preterm children and adolescents were recruited from the follow-up program of the two Institutions involved in the study, IRCCS Fondazione Stella Maris in Pisa and Fondazione IRCCS Istituto Neurologico Carlo Besta in Milano, according to the following criteria: (1) gestational age (GA) below or equal to 32 weeks; (2) absence of any major cerebral brain damage: normal results at neonatal cranial ultrasound (cUS) or periventricular increased echo density persisting less than 14 days and being less than or equal to grade I of De Vries’s classification^22^ and/or intreventricular hemorrage (IVH) grade I according to Papile et al.^23^; (3) global IQ within normal limits, (4) absence of main ocular anomalies including cataract, optic atrophy and retinopathy of prematurity and (5) age ranging from 12 to 17 years. All included patients were assessed using a validated routine protocol that involved repeated neonatal cranial ultrasound (cUS) performed weekly upon admission to the Neonatology Unit, continuing until discharge, and again at 1 and 3 months of corrected age. Additionally, all preterm participants underwent a neurological examination to exclude specific neurological signs at the time of recruitment. Furthermore, all preterm infants in the study were closely monitored for neurodevelopmental outcomes up to the age of two years, during which no issues were observed. Full term children and adolescents for the control group were recruited from the local secondary school, and matched for gender and age. An a-priori minimum sample size calculation was performed to be sure to enroll a sufficient number of subjects. The minimum sample size per group (one-tailed hypothesis) was 30.

The final cohort consisted of 30 preterm-born adolescents (mean age: 14.2 years, SD: 1.58, range: 12 years–16 years 6 months; mean GA 28.9 weeks, SD: 2.2 weeks, range 25–32; mean birth weight 1097 g, SD: 327 g, range 600–1820; 17 males) and 34 term-born adolescents (mean age: 14.5 years, SD: 1.8, range: 12 years 3 months–16 years 8 months; 19 males) which served as controls. The two groups did not differ according to IQ scores (preterm: 103; SD = 12.6; full-term: 103; SD = 13,28).

All subjects (preterm and controls) were administered a psychophysical test known as Form Coherence Task (modified version of Atkinson et al., 2006^24^), together with an IQ evaluation and a battery of well-standardized neuropsychological tests suitable for investigating ventral stream functioning.^11^ The battery included the Street Completion Test^25^ and the first part of the Visual Object and Space Perception (VOSP) battery.^26,27^ In addition, the second part of the VOSP battery was administered, to assess dorsal stream functioning, but it is not the focus of the present analyses.

### Psychophysical test

Detailed information about the Form coherence task can be found elsewhere.^11^ Briefly, the stimulus consisted of a static array of randomly orientated short line segments containing a target area on one side of the display where segments were oriented tangentially to form concentric circles. The proportion of tangentially oriented (coherent) line segments, amongst the randomly oriented noise segments in the target area, defined the coherence value for each trial. Participants were required to locate the target regions, which were presented in either the left or the right half of the display. In the testing phase, the initial coherence level on each task was set at 100% and was varied according to a modified version of the two-up/one-down staircase procedure.^24^ Starting at 100%, coherence was decreased stepwise between each trial, and the level at which the first incorrect response occurred formed the starting point for four reversals. The threshold was defined as the mean coherence level during the last four reversals. Lower raw scores corresponded to better performances.

### Street completion test

Objects made of black blotches representing parts of objects had to be recognized, therefore participants had to group the parts into a recognizable object. Eleven black and white pictures were showed in a fixed sequence, and the subject had to name what he thought he saw. Correct answers (CA) and incorrect answers (wrong recognitions, IA) were analyzed in the present study.

### Visual Object and Space Perception (VOSP) battery

Detailed information about the VOSP battery can be found elsewhere.^11^ Briefly, the battery includes object recognition (incomplete letters, silhouettes, object decision, progressive silhouettes) and space perception (dot counting, position discrimination, number location, cube analysis) tasks. The VOSP object recognition and space perception tasks are suitable for investigating the ventral and dorsal streams, respectively. Before performing these tasks, participants were screened with a Shape Detection Task in which they had to detect an ‘X’ in noise. The stimuli were presented as black-and-white pictures and drawings; the required response was single-word naming or pointing to the correct item. No drawing or copying was required.

Subjects were administered the whole battery, but in the present study, we analyzed only the object recognition tasks (incomplete letters, silhouettes, object decision and progressive silhouettes). For letters, silhouettes, and object decision tasks, the raw score was calculated by the sum of the correct answers (i.e. higher is better). For the progressive silhouette task the score was calculated by the sum of the figures that each subject had to see before recognizing the object (lower is better).

### Statistical analyses

Statistical analyses were performed using SPSS Statistics 20 software.^28^ The T-test for independent samples was used to compare results on IQ, GA, birth weight, Form coherence task, Street completion task and VOSP tasks in male and female preterm adolescents. The T-test for independent samples was also performed separately in males and females to evaluate differences in each task according to prematurity. A multivariate analysis of variance was performed to test the interaction between sex and GA in predicting performance on the Form coherence task, Street completion task and VOSP object recognition battery, in which the dependent variables were the scores obtained on the different tasks and the two group factors were sex (male vs female) and GA (<28 weeks vs ≥ 28 weeks). Finally, Spearman’s correlation was calculated separately in males and females in preterm and full-term adolescents, to test the association between Form coherence, Street completion and VOSP object recognition tasks. A *p*-value below 0.05 (one-tailed) was interpreted as significant.

All the analyses were performed using standardized z-scores to account for the deviation from the average and to correct for the age of the subjects.

## Results

**Fig. 1:**
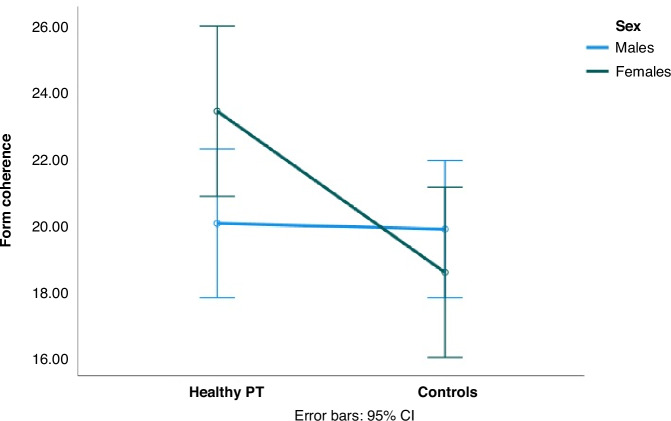
Form coherence thresholds in healthy preterms and controls according to sex. Form coherence Form Coherence thresholds, PT preterm, m male, f female, CI confidence interval.

Preterm and full-term groups were comparable according to IQ, age, sex distribution, and neuropsychological performances. The preterm group presents a higher threshold in the Form perception task indicating lower performance (Table 1, Fig. 1).

**Table 1:** Distribution of clinical and psychophysiological variables in full-term and healthy premature adolescents

	Preterm *n* = 30 Mean (SD)	Full-term *F* = 34 Mean (SD)	T-test/Chi-square† *p*-value
IQ	103 (12.6)	103 (13.28)	*T* = 0.083, *p* = 0.467
Age (years)	14.2 (1.6)	14.5 (1.8)	*T* = −0.727, *p* = 0.235
Male sex	21 (70%)	17 (50%)	Chi^2^ = 0.679, *p* = 0.436
Form coherence task	21.5 (4.7)	19.3 (4.8)	*T* = 1.810, *p* = 0.038
VOSP_Incomplete Letters	19.5 (0.57)	19.5 (0.66)	*T* = −0.328, *p* = 0.372
VOSP_Silhouette	18.5 (3.9)	18.9 (3.4)	*T* = −0.322, *p* = 0.374
VOSP_Object decision	16.6 (2.2)	16.7 (1.8)	*T* = −0.218, *p* = 0.414
VOSP_Progressive silhouette	10 (2.4)	9.8 (2.3)	*T* = 0.404, *p* = 0.344
Street_CA	8.4 (1.6)	8.1 (1.4)	*T* = 0.647, *p* = 0.260
Street_IA	2.2 (1.6)	2.2 (1.2)	*T* = −0.156, *p* = 0.438

*SD* Standard deviation, *CA* correct answers, *IA* Incorrect answers, *IQ* Intelligence Quotient, *p* *<* 0.05 one tailed.

In the control group, no differences were present in any of the considered variables according to sex. In the preterm group, GA were comparable between males and females, while birth weight was reduced in females (Table 2), as is consistently described in the literature.^29^

**Table 2:** Sex/gender distribution of clinical, psychophysiological and neuropsychological variables in healthy premature adolescents

	Males *n* = 17 Mean (SD)	Females *F* = 13 Mean (SD)	T-test† *p*-value
IQ	103 (10)	104 (14)	*T* = −0.312, *p* = 0.757
GA, weeks	29.5 (2.3)	28 (1.9)	*T* = 1.941, *p* = 0.062
Birth weight, grams	1200 (339)	950 (254)	*T* = 2.155, *p* = 0.040
Form coherence task	20 (4.08)	23 (4.80)	*T* = −2.078, *p* = 0.047
VOSP_Incomplete Letters	19.5 (0.52)	19.4 (0.7)	*T* = 0.533; *p* = 0.299
VOSP_Silhouette	18 (4.2)	19.3 (3.4)	*T* = 0.905, *p* = 0.374
VOSP_Object decision	16.4 (2.8)	16.8 (1.4)	*T* = −0.555, *p* = 0.583
VOSP_Progressive silhouette	10.6 (2.4)	9.3 (2.2)	*T* = 1.514, *p* = 0.142
Street_CA	8.2 (1.8)	8.7 (1.2)	*T* = −0.781, *p* = 0.442
Street_IA	2.4 (2)	1.9 (1.1)	*T* = 0.728, *p* = 0.473

†T-test for independent samples; significant results are considered at *p* ≤ 0.05, two tailed. *SD* standard deviation, *GA* gestational age, *CA* correct answers, *IA,* Incorrect answers, *IQ,* Intelligence Quotient.

### Psychophysical test

Preterm females obtained a poorer psychophysiological performance on the form coherence task than preterm males (Table 2, Fig. 1), and the effect of sex remained significant if considering GA in the multivariate model as second factor (Table 3). No other significant differences in neuropsychological tests were present in the preterm group according to sex or GA.

**Table 3:** Effect of sex and gestational age on psychophysical and neuropsychological tasks

	Males Mean (SD)	Females Mean (SD)	Effect of Sex	Effect of GA	Sex - GA interaction
Form; ≤28 weeks	*N* = 6 19.1 (3.9)	*N* = 8 23 (5.5)	*F* = 4.599, *p* = 0.042	*F* = 0.504; p = 0.482	*F* = 1.551; *p* = 0.225
Form; >28 weeks	*N* = 11 21 (4.3)	*N* = 5 24 (3.9)			
VOSP Incomplete letters; ≤28 weeks	19.3 (0.52)	19.5 (0.76)	*F* = 0.270, *p* = 0.608	*F* = 0.006; *p* = 0.941	*F* = 1.590; *p* = 0.219
VOSP Incomplete letters; > 28 weeks	19.6 (0.52)	19.2 (0.45)			
VOSP Silhouette; ≤28 weeks	19 (3.58)	20.5 (3.2)	*F* = 0.260, *p* = 0.615	*F* = 2.553; *p* = 0.123	*F* = 0.260; *p* = 0.615
VOSP Silhouette; > 28 weeks	17.4 (4.62)	17.4 (3.05)			
VOSP Object decision ; ≤28 weeks	16.33 (3.08)	16.88 (1.36)	*F* = 0.269, *p* = 0.609	*F* = 0.000, *p* = 0.996	*F* = 0.006, *p* = 0.938
VOSP Object decision; > 28 weeks	16.4 (2.76)	16.8 (1.64)			
VOSP Progr Silhouette; ≤28 weeks	9.17 (1.47)	9.12 (1.81)	*F* = 1.270, *p* = 0.270	*F* = 2.657, *p* = 0.116	*F* = 1.164, *p* = 0.291
VOSP Progr Silhouette; >28 weeks	11.5 (2.49)	9.6 (2.88)			
Street_CA; ≤28 weeks	7.88 (2.48)	8.71 (1.25)	*F* = 0.699, *p* = 0.412	*F* = 0.122, *p* = 0.730	*F* = 0.277, p = 0.603
Street_CA; >28 weeks	8.4 (1.43)	8.6 (1.34)			
Street_IA; ≤28 weeks	2.83 (2.64)	2.0 (1.15)	*F* = 1.738, *p* = 0.399	*F* = 0.500, *p* = 0.486	*F* = 0.163, *p* = 0.690
Street_IA; >28 weeks	2.1 (1.52)	1.8 (1.09)			

*GA* Gestational Age, *CA* correct answers, *IA* incorrect answers. Significant results are considered at *p* ≤ 0.05

Significantly poorer psychophysiological performance was evident when comparing preterm with full-term females, while there were no significant differences between the two male groups (Table 4). No significant differences in neuropsychological accuracy is present when comparing preterm with full-term boys or girls (Table 4). Form coherence tasks may be more sensitive to evidence preterm vulnerability in females, even in presence of comparable neuropsychological accuracy.

**Table 4:** Differences between Preterm and Full-term males and females on the Form coherence task and neuropsychological performances†

	Preterm Mean score (SD)	Full-term Mean score (SD)	T-test; *p*-value
Males	*N* = 17	*N* = 20	
Form Coherence	20 (5.2)	19.8 (4.7)	*T* = 0.718; *p* = 0.905
Street_CA	8.19 (1.83)	8.10 (1.51)	*T* = 0.168; *p* = 0.438
Street_IA	2.38 (1.96)	2.14 (1.28)	*T* = 0.435; *p* = 0.666
Females	*N* = 13	*N* = 14	
Form Coherence	23 (4.80)	18 (4.80)	*T* = 2.525, *p* = 0.019
Street_CA	8.67 (1.23)	8.23 (1.23)	T = 0.883; *p* = 0.386
Street_IA	1.92 (1.08)	2.38 (1.19)	*T* = -1.024; *p* = 0.317

†T-test for independent samples; results are considered significant at *p* ≤ 0.05, two tailed.

*SD* standard deviation, *IA* Incorrect answers, *CA* correct answers, *Street_CA*, more is better, *Street_IA* less is better, Form Coherence: less is better.

### Correlation between Form coherence and VOSP tasks

**Fig. 2:**
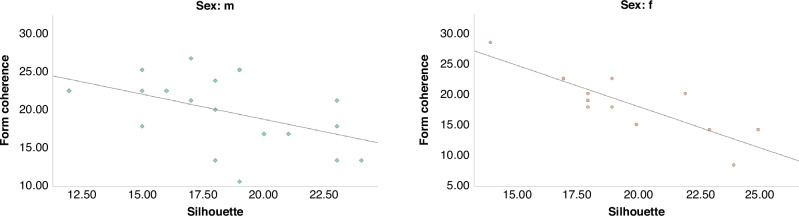
Correlations between Form coherence test and VOSP tasks in healthy controls. Form coherence Form coherence raw score, Silhouette Silhouette test raw score, m male, f female.

In the control group, non parametric correlations were present between Form coherence task and VOSP “Letters” (Rho *=* −0.437, *p* *=* 0.006) and “Silhouette” (Rho *=* −0.651, *p* *<* 0.001) tasks, indicating that a lower psychophysical threshold for the form perception is related to higher neuropsychological accuracy. Further investigating these results differentiating males and females controls (Fig. 2), we found that the correlation between Form coherence and VOSP “Silhouette” task was present in both males (Rho *=* −0.502, *p* *=* 0.012) and females (Rho = 0.824, *p* *<* 0.001), while the correlation between Form coherence and VOSP “Letters” was present only in males (Rho *=* −0–467, *p* *=* 0.019; females: Rho *=* −0.270, *p* *=* 0.186).

In the preterm group, no significant correlation was present between Form coherence and VOSP visual tasks, neither in the total group, nor in the two sex groups analyzed separately.

**Fig. 3:**
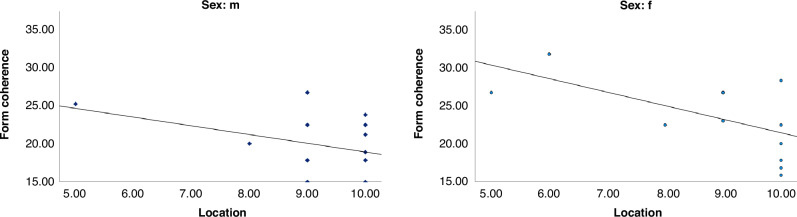
Correlations between Form coherence test and VOSP tasks in healthy preterm children. Form coherence Form coherence raw score, location number location test raw score, m male, f female.

In light of the results obtained from the visual tests, we have repeated the analyses on the association between the Form coherence and spatial task “number location”, which in the initial study was present only in the preterm group and not in the control group,^11^ separately for male and female preterm adolescents. The correlation was confirmed in preterm females (Fig. 3, Rho *=* −0.602, *p* *=* 0.015) but not in males (Rho *=* −0.208, *p* *=* 0.220).

## Discussion

The present study expands the previous results about functional vulnerability of the ventral and dorsal stream in adolescents born between 25 and 32 weeks and without neuromotor, sensory and neurological sequelae, providing further insights about a possible sex specificity.

First, we described a preterm-born female’s poorer form perceptual threshold in the psychophysical correlates of visuo-cognitive domain. In the whole sample, prematurity, but not sex, was related to lower psychophysiological performance. However, when separating the two sex groups, we found a preterm vulnerability only in females, with lower performance in the form coherence task when compared to full-term girls and both male’s groups. In the presence of prematurity, a greater female vulnerability in central visuo-cognitive processing seems to be more evident in our sample. Of notice, the female vulnerability emerged in the physiological threshold, but not in neuropsychological accuracy measured at visual object recognition and street completion tasks, and remained significant also when controlling for the level of prematurity (e.g. GA ≤ 28 weeks vs. > 28 weeks).

Previous studies have explored the cognitive outcomes of prematurity, suggesting that males are more susceptible to increased neurodevelopmental and cognitive deficits compared to preterm females. These deficits may include greater impairments in speech, language, academic achievement, and social functioning.^30–32^ Some recent studies have also investigated the mechanisms that contribute to the apparent resilience of the female brain using connectivity analyses.^34,35^ These studies have occasionally included children with brain lesions and neurological conditions associated with prematurity that were identified during the neonatal period^30,33,35^ that could confound the results. Sex comparisons in the current sample of school age children without prematurity-related diseases, revealed a trend opposite to the so-called *male disadvantage* hypothesis observed in prematurity-related developmental conditions. This aligns partially with the findings of a recent meta-analysis on former preterm children, which did not find significant male-biased or female-biased effects of prematurity on cognitive domains.^7^ It’s worth noting that this meta-analysis included approximately 13 studies assessing cognitive abilities primarily through IQ measurements, without specific focus on visuo-perceptual and visuo-spatial abilities. Additionally, the inclusion of children with prematurity-related disorders was not discussed.

The present findings are consistent with and partially extend the discussion presented by Guzzetta et al.,^14^ indicating that deficits in the ventral stream (form perception) are associated with periventricular brain damage. In our sample of neurologically healthy preterm adolescents, we did not observe sex differences in neuropsychological performance, but we did find differences in the form perceptual threshold, linked to ventral stream functioning. Therefore, using measures that are more sensitive to psychophysical differences could effectively reveal mild dysfunctions even in former preterm adolescents with good cognitive and clinical neurological outcomes. Further studies excluding children with neurological problems of prematurity are necessary to obtain more consistent brain-behavioral correlation explaining sex differences in preterm-born children in the visuo-perceptual development.

The correlation between the psychophysical measure of perceptual threshold and neuropsychological performances present in both healthy preterm and full-term subjects confirms that the Form Coherence task may be an effective tool to investigate visuo-cognitive domain in healthy premature adolescents and that the VOSP battery may be a feasible tool to investigate the ventral stream functioning with some sensitiveness to sex differences in premature children and adolescents. Moreover, the correlation between Form coherence test and spatial task “number location”, which in the initial study was present only in the preterm group and not in the control group,^11^ was confirmed in preterm females but not in males. The presence of a correlation between the form coherence – more ventral task – and VOSP’s subtest investigating not only ventral (silhouettes), but also dorsal stream (number location), expands the previous evidence about functional interaction between the two visual streams, as suggested by Milner,^36^ that can be understood not as independent processes but based on more balanced approach to inter-stream communication. This correlation was evident in the whole sample of preterm adolescents,^11^ but when we investigated it separately for males and females, it revealed that the effect was present only in preterm females, suggesting different functioning of the ventral and dorsal stream according to sex.

Taken together, these results could suggest that, in females born preterm without clinical neurological sequelae, with preserved cognitive and neuropsychological abilities, the efficiency of the psychophysical perceptual threshold is reduced, but not related to the neuropsychological performance. Females may implement compensation strategies to achieve good performance regardless of the perceptual threshold.

Although this is an exploratory study that needs to be replicated in larger samples to strengthen the statistical power, its findings are in the direction of a possible sex-specific outcome in the visuo-spatial and visuo-perceptual domain in preterm children. In the present study, we minimized the influence of neurological complications by selecting children who had normal neonatal cranial ultrasound (cUS) and demonstrated good intellectual and neurological outcomes. Neonatal cUS, along with subsequent neurological assessments, are considered the gold standard in clinical practice for screening neurological complications in preterm children,^37,38^ showing higher sensitivity than neonatal MRI.^39^ However, relying solely on neonatal cUS and follow-up neurological exams may not be sufficient to confirm the absence of brain disorders in older children. Follow-up structural MRI, including advanced techniques, at the time of neuropsychological testing would be more appropriate to definitively confirm the absence of brain lesions.

## Conclusions

Studies about sex differences in the outcome of premature children are heterogeneous and lack methodological control for male disadvantage related to neurological risk. Male-female differences in the recovery and long-term outcomes of preterm children are influenced by several potential mediators, such as major and minor neurological diseases, environment and brain structure-connectivity. Our original data on preterm adolescents with normal cognitive abilities and clinical neurological follow-up who were administered visuo-perceptual tasks reveal a female disadvantage in the visuo-cognitive domain, although these results need to be replicated in a larger sample.

## Data Availability

The data that support the findings of this study are available on request from the corresponding author.
